# A simple-to-use nomogram for predicting prolonged mechanical ventilation for children after Ebstein anomaly corrective surgery: a retrospective cohort study

**DOI:** 10.1186/s12871-022-01942-9

**Published:** 2023-01-14

**Authors:** Qiao Liu, Qipeng Luo, Yinan Li, Xie Wu, Hongbai Wang, Jiangshan Huang, Yuan Jia, Su Yuan, Fuxia Yan

**Affiliations:** 1 Department of Anesthesiology, Fuwai Hospital, National Center of Cardiovascular Diseases, Chinese Academy of Medical Sciences and Peking Union Medical College, 167 Beilishi Road, Xicheng District, Beijing, 100037 China; 2 Department of Pain Medicine, Peking University Third Hospital, Beijing, China

**Keywords:** Ebstein anomaly (EA), Prolonged mechanical ventilation (PMV), Pediatric cardiac surgery, Risk factor, Nomogram

## Abstract

**Background:**

Prolonged mechanical ventilation (PMV) after pediatric cardiac surgery imposes a great burden on patients in terms of morbidity, mortality as well as financial costs. Ebstein anomaly (EA) is a rare congenital heart disease, and few studies have been conducted about PMV in this condition. This study aimed to establish a simple-to-use nomogram to predict the risk of PMV for EA children.

**Methods:**

The retrospective study included patients under 18 years who underwent corrective surgeries for EA from January 2009 to November 2021. PMV was defined as postoperative mechanical ventilation time longer than 24 hours. Through multivariable logistic regression, we identified and integrated the risk factors to develop a simple-to-use nomogram of PMV for EA children and internally validated it by bootstrapping. The calibration and discriminative ability of the nomogram were determined by calibration curve, Hosmer-Lemeshow goodness-of-fit test and receiver operating characteristic (ROC) curve.

**Results:**

Two hundred seventeen children were included in our study of which 44 (20.3%) were in the PMV group. After multivariable regression, we obtained five risk factors of PMV. The odds ratios and 95% confidence intervals (CI) were as follows: preoperative blood oxygen saturation, 0.876(0.805,0.953); cardiothoracic ratio, 3.007(1.107,8.169); Carpentier type, 4.644(2.065,10.445); cardiopulmonary bypass time, 1.014(1.005,1.023) and postoperative central venous pressure, 1.166(1.016,1.339). We integrated the five risk factors into a nomogram to predict the risk of PMV. The area under ROC curve of nomogram was 0.805 (95% CI, 0.725,0.885) and it also provided a good discriminative information with the corresponding Hosmer-Lemeshow *p* values > 0.05.

**Conclusions:**

We developed a nomogram by integrating five independent risk factors. The nomogram is a practical tool to early identify children at high-risk for PMV after EA corrective surgery.

**Supplementary Information:**

The online version contains supplementary material available at 10.1186/s12871-022-01942-9.

## Background

Ebstein anomaly (EA) is a rare and complex congenital heart disorder with an incidence of 1 per 200,000 live births [1]. It is characterized by the primary displacement of septal or posterior leaflets of the tricuspid valve and a variable amount of atrialization of the right ventricle (RV) [2], mild to severe tricuspid regurgitation (TR) is almost invariably present [3]. Corrective surgery is one of the therapeutic options for EA patients, with a wide variation of reported mortality (from 2.5 to 31%) [4]. Despite the improvements in medical technique and experience, there are still many challenges for perioperative management of EA patients even after successful corrective surgeries, such as low cardiac output syndrome, right heart failure, arrhythmias, prolonged mechanical ventilation (PMV), kidney injury, hepatic injury and others [3, 5], which can significantly increase the risk of perioperative management and affect the long-term prognosis of patients.

Patients often need mechanical ventilation after pediatric cardiac surgery, although mechanical ventilation is a supportive treatment for them especially for critical children, PMV is always associated with lung injury. Several mechanisms are involved in ventilator induced lung injury, including barotrauma, volutrauma, atelectrauma and ventilator induced diaphragmatic dysfunction [6, 7]. The development of ventilator induced diaphragmatic dysfunction is always a vicious circle [8], this process may be mediated by oxidative stress and could develop within the first day of mechanical ventilation [9]. For pediatric cardiac surgery, earlier studies have proved that patients with PMV are related to a higher mortality compared with non-PMV, and varied widely from 6.6 to 34% [10–13]. Now, the research about PMV mainly focus on the postoperative complications, hospital stay time and medical resource utilization [14–17]. Some medical centers have taken the postoperative mechanical ventilation as a quality metric for pediatric cardiac surgery [18]. Hence, early and accurate identification of children at high-risk for PMV could assist clinicians to enact better individualized medical management, improve prognosis and allocate medical resources rationally. However, because of the rarity of the EA disease, there is no established scoring model used to identify high-risk PMV for EA children. The purpose of the present study was to evaluate the incidence rate and independent risk factors of PMV for EA children, and construct a nomogram to early identify children at high risk for PMV after EA corrective surgery.

## Methods

The study report adheres to Strengthening the Reporting of Observational Studies in Epidemiology (STROBE) statement and the details are seen in Additional file 1. The retrospective study was approved by Ethics Committee of the Chinese Academy of Medical Sciences Fuwai Hospital (NO.2022–1647) and waived the need for informed consent. All the methods in study have been carried out in accordance with our institutional regulations and guidelines. The procedures performed in study are complied with the ethical standards of the institutional research committee, and with the 1964 Helsinki Declaration and its subsequent amendments. Patients under 18 years of age who underwent EA corrective surgery in Fuwai hospital from January 2009 to November 2021 were included. Patients who met the following criteria were excluded:1) patients with missing data with postoperative MV time; 2) patients with confounding factors that may impact postoperative MV time, including preoperative tracheal intubation or tracheotomy, preoperative pulmonary infection, previous palliative surgery history or additional undergoing palliative surgery, combined with complex cardiac malformations (corrected transposition of a great artery, pulmonary atresia, single ventricle, endocardial cushion defect, coronary artery fistula); 3) surgery associated factors including emergency surgery, surgical re-exploration and bedside thoracotomy; 4) postoperative complications including death, tracheotomy, arrhythmia, thrombosis and extracorporeal membrane oxygenation implantation. The EA surgical indications included severe TR, cyanosis, dyspnea, right heart failure, progressive cardiac hypertrophy, a cardiothoracic ratio (C/R) more than 0.65, combined with other cardiac malformations, and poorly controlled tachyarrhythmias [19]. After EA corrective surgery, patients were transferred to the pediatric intensive care unit (ICU). The extubation standards of ICU were as follows: spontaneous respiration recovery, hemodynamic stabilization, adequate consciousness, airway reflexes completely recovery and manageable airway secretions [20]. The decision to extubate or reintubate was made by anesthesiologist, intensivist and attending surgeon. About reintubation, we recorded the cumulative mechanical ventilation time.

**Fig. 1:**
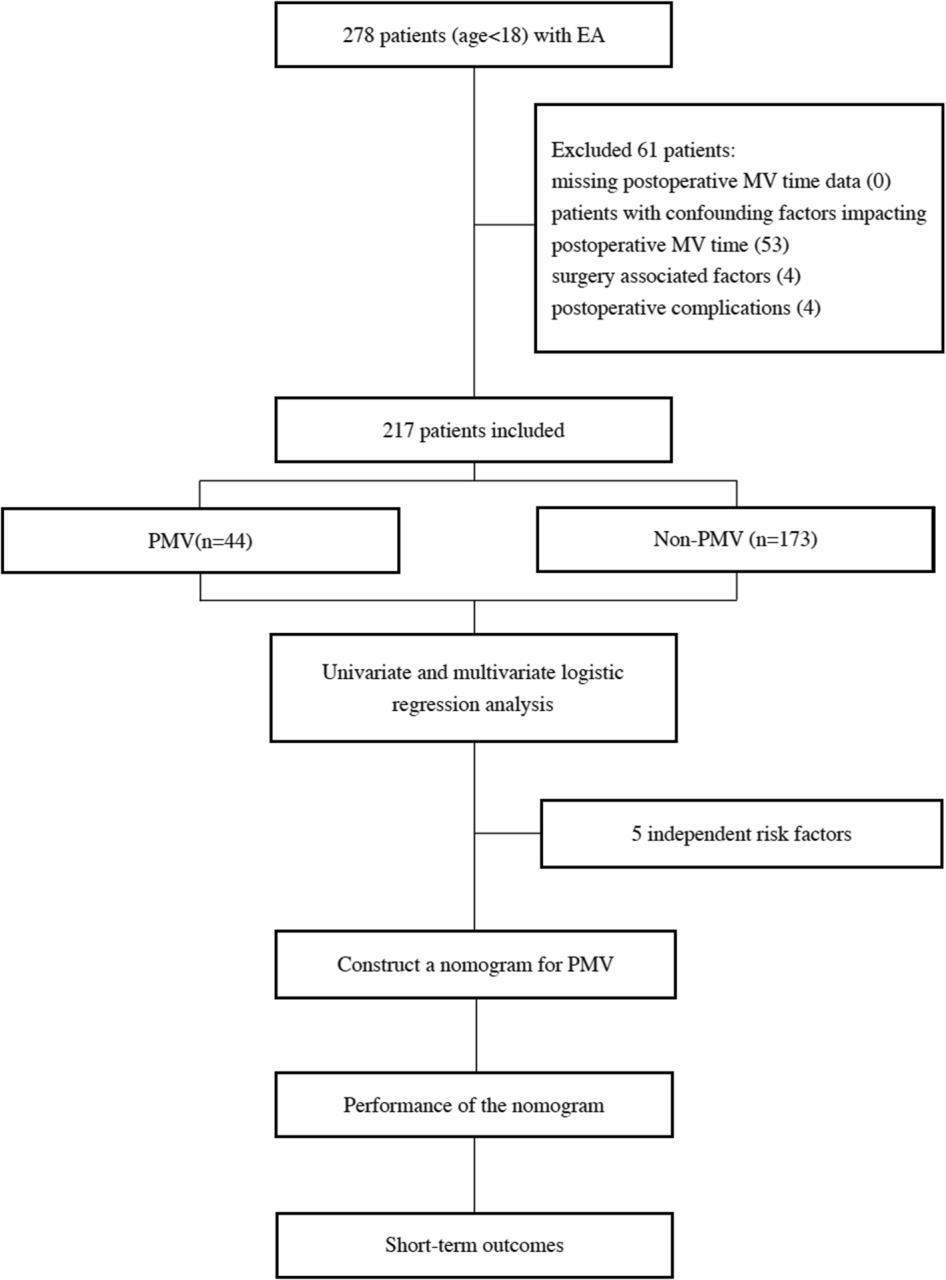
The Flowchart of the study. EA, Ebstein anomaly; PMV, prolonged mechanical ventilation

The workflow of the study is depicted in Fig. 1. Based on The Society of Thoracic Surgeons [17], patients with postoperative mechanical ventilation longer than 24 hours were included in the PMV group, and the others in the Non-PMV group. We compared the associated clinical data between the two groups, after univariable and multivariable logistic regression analyses, the independent risk factors for PMV were screened out. The independent risk factors of PMV in the multivariable analysis were integrated to construct a nomogram for predicting PMV and internally validated it by bootstrapping. The performance of the model was evaluated by calibration curve, Hosmer-Lemeshow goodness-of-fit test and ROC curve. Finally, we compared the short-term outcomes of the two groups. About the multivariable logistic model, we conducted a sample size calculation that could produce a small prediction error in the target population [21]. The calculation is available online (https://mvansmeden.shinyapps.io/BeyondEPV/). n is the sample size, ϕ is the anticipated outcome proportion (≤0.5), P is the number of candidate predictor parameters (≤30); MAPE refers to the average error of the allowable estimated outcome probability in the model. Generally, the recommendation for MAPE is no larger than 0.05. However, considering that ebstein anomaly is a rare disease with a small sample size, we set MAPE as 0.1, P as 9 and ϕ as 0.2; finally, this study requires at least 180 participants (about 36 expected events).

For data collection, we have three independent medical students recorded the clinical data and postoperative short-term outcomes from admission to discharge through electronic medical records, and two independent researchers checked the data. The preoperative data were collected as follows: age, gestational age, sex, weight, height, body mass index, diagnosis, peripheral oxygen saturation (SpO2), New York Heart Association Classification, C/R, arrhythmia, cardiac defects, echocardiography data, and blood routine test. The preoperative SpO2 was collected when patient inhaled the air at admission to hospital. Carpentier’s classification was used to categorize the anatomy of EA [22]. The degree of TR was graded as none, mild, moderate, moderate-to-severe and severe on echocardiographic analysis. This study is a pediatric cohort, the left ventricular end-diastolic diameter z-score (LVEDDz) was used to present left ventricular end diastolic diameter. We applied the Z-scores indexed to BSA to calculate LVEDDz and the calculation is available online (http://parameterz.blogspot.com/2008/09/m-mode-z-scores.html.) [23]. Intraoperative data included cardiopulmonary bypass (CPB) time, aortic cross-clamp time, minimum temperature during CPB, anesthetic, blood loss, infusion volume, mean arterial blood pressure (MAP), and central venous pressure (CVP). The MAP and CVP were the first measurement after 30 min of admission into pediatric ICU.

We chose PMV as the primary outcome and in-hospital outcomes as the secondary outcome. The in-hospital outcome included the duration of ICU stay, hospital stay and postoperative hospital stay, acute kidney injury (AKI), acute hepatic injury (AHI), and hospitalization costs. The hospital stay time was defined as the duration from admission to discharge, and the postoperative hospital stay time was defined as the duration from the end of surgery to discharge. AKI was defined as postoperative creatinine level more than 1.5-fold the baseline level [24]. AHI was defined as postoperative aspartate aminotransferase (AST) or alanine aminotransferase more than 2 times the upper limit. Due to the CPB associated hemolysis may induce AST to increase, an increased AST within 24 hours after operation was not considered to be AHI [25, 26].

### Statistical analysis

The normally distributed continuous variables were described as the means ± standard deviation and compared by the Student’s t-tests. For non-normally distributed continuous variables, they were presented as the median with 25–75 percentiles and analyzed by Mann-Whitney U tests. Categorical variables were expressed as absolute (n) and relative (%) frequency, they were analyzed by Chi-squared test. We performed univariable and multivariable logistic regression analyses to obtain the independent risk factors for PMV. Collinearity relationships between risk factors were conducted before multivariate analysis by tolerance and VIF. Tolerance < 0.1 or VIF > 10 indicated that collinearity exist between variables. All variables with *P* < 0.1 in univariable regression and the clinically relevant variables were put into a multivariable regression model by forward variable selection. We assessed the calibration and discrimination ability of the nomogram by the calibration curve, Hosmer-Lemeshow goodness-of-fit test and ROC curve. DeLong’s method was used to compare the areas under ROC curves (AUCs) [27]. To reduce confounding effect of age and surgical procedure, we conducted the subgroup analysis of age ≤ 6 years old and TVP groups*. P* value< 0.05 was considered statistically significant. All statistical analysis was conducted in SPSS software version 25 (IBM, Armonk, NY, USA) and R software (version 3.2.0).

## Results

### Basic characteristics

There were 278 children who underwent EA corrective surgeries at Fuwai Hospital in the past 13 years. We excluded patients with a) missing postoperative MV time data (*n* = 0); b) confounding factors impacting postoperative MV time (*n* = 53); c) surgery associated factors(*n* = 4) and d) postoperative complications (*n* = 4). Finally, 217 patients were enrolled in our study, of which 44 patients were in the PMV group and 173 patients were in the Non-PMV group (Fig. 1). The median age at surgery of the whole cohort was 6.1 (3–11.7) years, 123 (56.7%) patients were male, and 30(13.8%) patients had a C/R greater than 0.65. Most patients had associated cardiac anomalies of which the most common were atrial septal defect and patent foramen ovale, which were respectively present in 81(37.3%) and 68 (31.3%) of the patients. With regard to the echocardiographic parameters, the median ejection fraction was 66%(62.5,70%), LVEDDz was − 1.87(− 2.79, − 1.06) and aortic annular diameter was 15 mm, TR more than moderate was present in 72.4% of patients and Carpentier type C or D was present in 20.3% of patients. All surgeries were performed under CPB, with a median CPB time of 107 (89–136) minutes and aortic cross-clamp time of 74 (62–94) minutes. Table 1 presents a summary of demographic data and perioperative variables of all patients. Compared with the Non-PMV group, patients in the PMV group had smaller weight and height, lower preoperative SpO2, larger C/R, longer CPB and ACC time, and higher CVP.

**Table 1:** The demographic and perioperative information in study children

Variable	Total *n* = 217	Non-PMV *n* = 173	PMV *n* = 44	*P value*
Demographics				
Age (years)	6.1 (3,11.7)	6.23 (3.13,11.92)	4.12 (2.02,10.74)	0.158
Gestational age (weeks)	40 (39,40)	40 (39.1,40)	40 (39,40)	0.756
male	123 (56.7%)	96 (55.5%)	27 (12.4%)	0.483
Weight (kg)	20 (14,41.5)	21 (14,44)	16 (11.38,31.93)	0.026
Height (cm)	115 (92,152)	118 (96,155)	104 (83,140.5)	0.049
BMI (kg/m^2^)	16.56 (15.22,19.15)	16.8 (15.27,19.41)	15.89 (14.81,18.61)	0.087
Preoperative data				
SpO2(%)	98 (96,99)	98 (96,99)	97.5 (92.25,100)	0.268
NYHA class (III/IV)	23 (10.6%)	16 (9.2%)	7 (15.9%)	0.2
C/R > 0.65	30 (13.8%)	19 (11%)	11 (25%)	0.016
WPW syndrome	29 (13.4%)	23 (13.3%)	6 (13.6%)	0.953
Associated cardiac defects				
ASD	81 (37.3%)	63 (36.4%)	18 (40.9%)	0.582
PDA	4 (1.8%)	2 (1.2%)	2 (4.5%)	0.136
PFO	68 (31.3%)	56 (32.4%)	12 (27.3%)	0.515
Ultrasound parameter				
LVEF (%)	66 (62.5,70)	66 (63,70)	66 (61,70)	0.884
TR > moderate	157 (72.4%)	121 (69.9%)	36 (81.8%)	0.117
Carpentier type C or D	44 (20.3%)	25 (14.5%)	19 (8.8%)	< 0.001
LVEDDz	−1.87(−2.79,-1.06)	−1.85(−2.76,-1.04)	−2(−3.04,-1.19)	0.537
RVAD (mm)	26 (19,32)	25 (19,31.5)	26 (19.25,32)	0.828
SLD (mm)	21 (14,27)	20 (14,27)	21.78 (11.75,27)	0.606
PLD (mm)	32.39 (21.5,42)	32.39 (20.5,43)	32.39 (22.75,36.75)	0.557
AOAD (mm)	15 (13,17.5)	15 (13,18)	13.5 (11.25,17)	0.058
Preoperative biomarkers				
WCC (10^9^/L)	7.85 (6.31,9.65)	7.81 (6.34,9.34)	8.05 (6.17,10.33)	0.562
RBC(10^12^/L)	4.81 (4.52,5.21)	4.83 (4.56,5.22)	4.74 (4.47,5.17)	0.396
PLT (10^9^/L)	285 ± 75.2	287.5 ± 72.1	275.5 ± 86.6	0.112
Hb(g/L)	136 (127,145)	136 (127,145)	133.5 (126.3143.8)	0.56
Hct (%)	40.1 (37.4,43.1)	40.2 (37.6,43.25)	39.95 (36.63,41.93)	0.336
CK-MB (IU/L)	18 (6.8,28)	18 (8.5,28)	15 (3.6,25.7)	0.19
hs-CRP (mg/L)	0.47 (0.14,1.19)	0.47 (0.14,1.23)	0.45 (0.15,1.13)	0.83
Intraoperative data				
TVR	7 (3.2%)	5 (2.9%)	2 (4.5%)	0.579
CPB time (min)	107 (89,136)	101 (87,133.3)	131.6 (107.2166.3)	< 0.001
ACC time (min)	74 (62,94)	70 (61,91.5)	86 (71.3102.3)	0.004
T_min_ (°C)	30.1 (29.9,31)	30.2 (29.8,31)	30 (30,31)	0.566
Dexamethasone	144 (66.4%)	121 (69.9%)	23 (52.3%)	0.027
Ulinastatin	77 (35.5%)	61 (35.3%)	16 (36.4%)	0.891
Infusion volume (ml)	90 (55,300)	90 (55,300)	80 (55,200)	0.838
Blood loss (ml)	60 (30,250)	70 (30,300)	30 (20,157.5)	0.017
CVP (mmHg)	6 (4.5,8)	6 (4,8)	7.5 (6,9)	0.005
MAP (mmHg)	56.9 ± 12.5	57 ± 12.3	56.3 ± 13.2	0.799

Quantitative variables are expressed as means ± SD or median (interquartile range); Categorical variables are expressed as frequency (percentage). *BMI* Body mass index, *SpO2* Peripheral oxygen saturation, *NYHA class* New York Heart Association Classification, *C/R* Cardiothoracic ratio, *WPW* Wolff-Parkinson-White, *ASD* Atrial septal defect, *PDA* Patent ductus arteriosus, *PFO* Patent foramen ovale, *LVEF* Left ventricular ejection fraction; *TR* Tricuspid regurgitation, *LVEDDz* LEFT ventricular end-diastolic diameter z-score, *RVAD* Right ventricular anteroposterior diameter, *PLD* Posterior leaflets displacement, *SLD* Septal leaflets displacement, *AOAD* Aortic annular diameter, *WCC* White blood cell, *RBC* Red blood cell, *PLT* Platelet, *Hb* Hemoglobin, *Hct* Hematocrit, *CK-MB* Isoenzyme of creatine kinase-MB, *hs-CRP* C-reactive protein, *TVR* Tricuspid valve replacement, *CPB* cardiopulmonary bypass, *ACC* Aortic cross-clamp, *T*_*min*_ The minimum temperature, *CVP* Central venous pressure, *MAP* Mean artery blood pressure

### Development of the nomogram

**Fig. 2:**
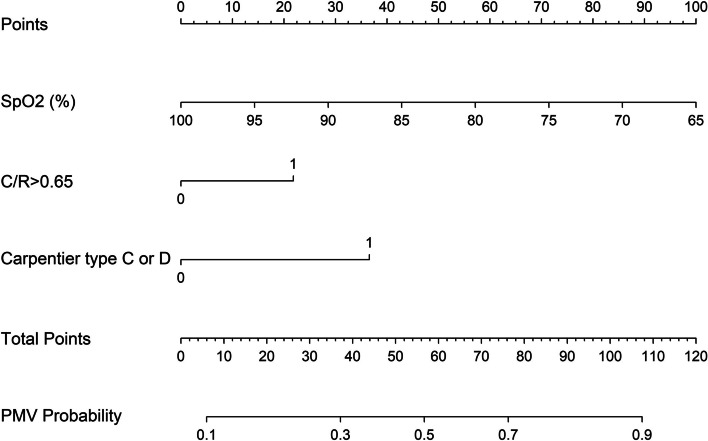
The Nomogram of prolonged mechanical ventilation**.** SpO2, peripheral oxygen saturation; C/R, cardiothoracic ratio; CPB, cardiopulmonary bypass; CVP, central venous pressure

The complete univariable logistic regression results were shown in Additional file 2. According to the univariable and multivariable regression results, we found that preoperative SpO2, C/R > 0.65, Carpentier type C or D, CPB time and CVP were independent risk factors for PMV. The odd ratios and 95% confidence intervals (CI) of risk factors for PMV are shown in Table 2. We conducted the multicollinearity test for the model: the tolerances are all more than 0.1 and VIF are all less than 10. There was no evidence of multicollinearity in the final multivariable model. Then we incorporated the independent risk factors into a nomogram of PMV in Fig. 2.

**Table 2:** Univariable and multivariable logistic regression analyses of risk factors for prolonged mechanical ventilation

Variable	Univariable analysis	*P* value	Multivariable analysis	*P* value
	OR (95%CI)		OR (95%CI)	
Age (y)	0.964 (0.902,1.03)	0.276		
SpO2 (%)	0.88 (0.816,0.948)	0.001	0.876 (0.805,0.953)	0.002
C/R > 0.65	2.702 (0.392,2.707)	0.019	3.007 (1.107,8.169)	0.031
Carpentier type C or D	4.499 (2.164,9.353)	< 0.001	4.644 (2.065,10.445)	< 0.001
AOAD (mm)	0.913 (0.825,1.01)	0.076		
CPB time (min)	1.015 (1.006,1.023)	< 0.001	1.014 (1.005,1.023)	0.002
ACC time (min)	1.012 (1.002,1.023)	0.019		
Dexamethasone	0.471 (0.24,0.924)	0.029		
CVP (mmHg)	1.202 (1.07,1.352)	0.002	1.166 (1.016,1.339)	0.029

*OR* Odds ratio, *CI* Confidence interval, *SpO2* Peripheral oxygen saturation, *C/R* Cardiothoracic ratio, *CPB* Cardiopulmonary bypass, *ACC* Aortic cross-clamp, *CVP* Central venous pressure

### Performance of the nomogram

**Fig. 3:**
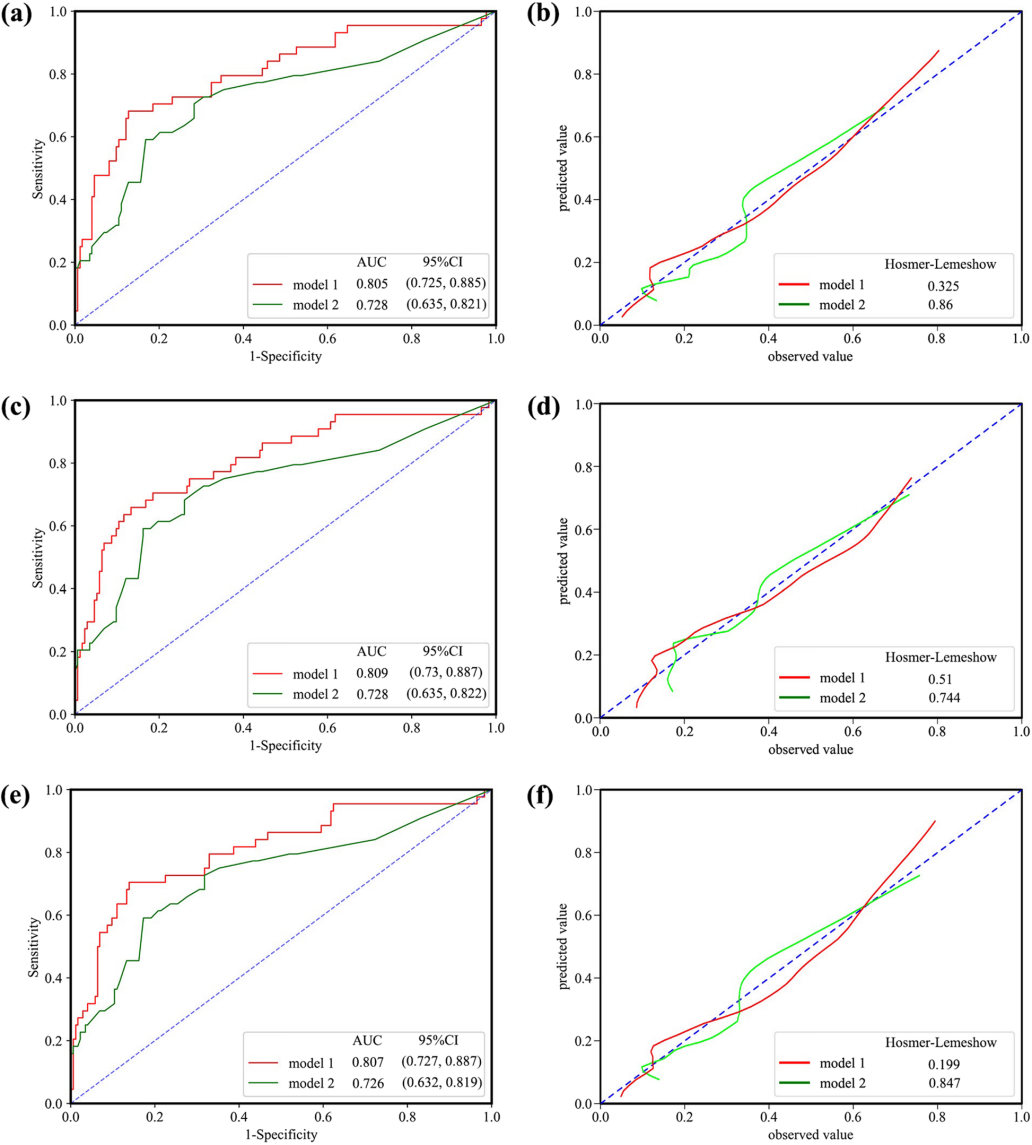
The performance of the nomogram for prolonged mechanical ventilation. **a** Receiver operating characteristic curve of the nomogram in all children. **b** The calibration curve of the nomogram in all children. **c** Receiver operating characteristic curve of the nomogram in the subgroup with age ≤ 6 years. **d** The calibration curve of the nomogram in the subgroup with age ≤ 6 years. **e** Receiver operating characteristic curve of the nomogram in the subgroup with tricuspid valvuloplasty. **f** The calibration curve of the nomogram in the subgroup with tricuspid valvuloplasty. Model 1: SpO2 + Carpentier type+C/R + CPB time + CVP; Model 2: SpO2 + Carpentier type+C/R. AUC, area under the receiver-operating characteristic curve*;* CI, confidence interval

The AUC of the PMV nomogram including perioperative variables was significantly increased by 0.0772 when adding intraoperative variables including CPB time and CVP (DeLong *p* < 0.05). The AUC of the final nomogram was 0.805 (95%CI, 0.725,0.885, *P* < 0.001) in the training cohort; and AUC was 0.833(95%CI 0.704,0868) for internal validation with bootstrapping. The nomogram also showed a good discriminative ability with the corresponding Hosmer-Lemeshow *p* values > 0.05 (*P* = 0.325). The ROC and calibration curves are displayed in Fig. 3a and Fig. 3b. The AUCs of nomogram in the subgroups with age ≤ 6 years old and TVP subgroup were 0.809 (95%CI, 0.73, 0.887, *P* < 0.001) and 0.807(95%CI,0.727,0.887, *P*<0.001), respectively (Fig. 3c,e). The corresponding calibration curves of subgroups are respectively shown in Fig. 3d and Fig. 3f.

### Short-term outcomes

The short-term outcomes for all patients are presented in Table 3. The median mechanical ventilation time for patients with PMV and Non-PMV were 26.5(24.5–63.75) and 9(6–14) hours, respectively. Patients with PMV were associated with a significantly higher incidence of reintubation. Although the incidence of AKI and AHI was not statistically different in the two groups, it was nominally higher in the PMV group. Patients in the PMV group had longer ICU stay time, longer postoperative hospital stay time and higher hospitalization costs.

**Table 3:** Short-term outcomes of the study children

Variable	Total *n* = 217	Non-PMV *n* = 173	PMV *n* = 44	*P value*
MV time (h)	11 (7,20)	9 (6,14)	26.5 (24.5,63.75)	< 0.001
AKI	57 (26.3%)	42 (24.3%)	15 (34.1%)	0.187
AHI	49 (22.6%)	36 (20.8%)	13 (29.5%)	0.216
Reintubation	2 (0.9%)	0 (0%)	2 (4.5%)	0.005
ICU stay time (d)	2 (1,3)	1 (1,2)	4 (2,6)	< 0.001
Hospital stay time (d)	14 (11,20)	13 (11,16.5)	20 (16.25,27.75)	< 0.001
Postoperative hospital stay time (d)	7 (6.3,9.6)	6.5 (6.2,7.8)	12 (9.4,14.9)	< 0.001
Hospital cost (¥ 1000)	66.7 (50.1,85.7)	60.3 (47.7,77.1)	99.2 (75.7120.2)	< 0.001

Quantitative variables are expressed as median (interquartile range); Categorical variables are expressed as frequency (percentage). *MV* Mechanical ventilation, *AKI* Acute kidney injury, *AHI* Acute hepatic injury, *ICU* Intensive care unit

## Discussion

Despite the great advances in perioperative management of cardiac surgery in recent years, PMV after pediatric cardiac surgery is still remains a major concern in postoperative medical care. In our cohort, approximately 20% of children were ventilated more than 24 hours after EA corrective surgery, which is consistent with earlier literature data (rang from 5 to 22%) [20, 28]. Studies in the setting of pediatric cardiac surgery showed that PMV is associated with adverse outcomes, such as pulmonary infection, renal failure, cardiac dysfunction, reintubation and other complications [14, 16, 29–31]. Compared with non-PMV group, patients in PMV group are linked to prolonged ICU stay time, increased hospital care costs, and higher postoperative mortality [20, 30, 32, 33]. The recent COVID-19 pandemic has shown that how important to rationally optimize the allocation of medical resources are. Early identification of high-risk PMV children would have potential economic effect for healthcare systems [11, 12, 29, 30]. Now several studies have reported associated risk factors and developed related prediction models for PMV [18, 34–36], but only few studies give attention to the specific cardiac defect type. Because of the heterogeneity of the disease, it’s difficult to put these models into clinical practice for a specific type of heart disease. The “China Birth Defect Prevention Report (2012)“showed that due to the relatively poor economic level in our developing country and to the fact that prenatal evaluation has not been fully popularized, congenital heart disease is still the most common birth malformation in China [37]. As the largest cardiovascular specialty hospital in China, we receive several EA children coming for surgery every year, thereby making a close attention for EA is meaningful in clinical practice. Until now no risk scoring model of PMV for EA children has been explored because of the rarity and various modes of presentation of EA. To our knowledge, we are the first to develop a nomogram to predict the risk of PMV for children with EA using routinely collected clinical variables.

About EA, due to tricuspid valve delamination failure, the septal and posterior leaflets are displaced distally to the true tricuspid annulus, long-term TR and the enlargement of the atrialized RV eventually cause volume overload, cardiomegaly and right heart failure [1, 4, 38]. Severe cardiomegaly can hinder pulmonary adequate expansion and impair pulmonary function [39, 40]. With the deterioration of cardiac function, the poor pulmonary dilatation and obvious cardiac enlargement would be found on chest X-ray [41, 42]. Despite advances in surgical techniques, most cardiac surgeries still need to be performed under CPB, it could activate the systemic immune response and produce multiple inflammatory mediators, increase capillary permeability and eventually result in extravasation of lung water [43–45]. This immune activation can decrease pulmonary compliance and lead to PMV [36, 46]. Hypoxia could contribute to lung metabolic dysfunction by activating oxidative stress and inflammatory response, eventually lead to pulmonary vascular endothelial tissue damage [47, 48], thereby we think that decreased SpO2 can be involved in the development of PMV. The anatomical structure of EA is very complex and changeable, Carpentier proposed a 4-grade classification to category the severity of EA’s anomaly by type A, B, C, and D [22]. Although Carpentier’s classification scheme could not be completely consistent with the severity of EA anatomy, it is widely used in clinical practice for simplicity and convenience, Carpentier C or D is usually combined with larger atrialized RV, more severe TR compared with type A or B [49]. Tobler found that the larger atrialized RV volume was related to the poor aerobic capacity [50]. We guessed that more severe EA anatomical type is related to decreased aerobic capacity, which can be involved in the development of PMV in EA patients.

In practical clinical work, clinicians tend to categorize patients as low or high risk to simplify work flow. The nomogram is featured by it can directly express the results of the complex statistical model, give the corresponding scores to the related factors and calculate the predicted probability of the event. Considering the underlying physical difference of children between PMV group and Non-PMV group maybe contribute to the development of PMV, we collected perioperative clinical data to identify the independent risk factors of PMV, and further to establish a simple-to-use nomogram that can provide a relatively accurate and objective prediction of PMV for children after EA surgery. The PMV nomogram showed a good but not excellent performance with an AUC of 0.805, it was comprehensible as we excluded patients with known risk factors to eliminate confounding factors*.* Due to the relatively poor economic status of our country may induce patients to come to the hospital at an older age, and considering our cardiac surgeons prefer to conduct TVP for patients when technology is feasible, this nomogram was applied into the subgroups with age ≤ 6 years old and TVP groups, it still showed acceptable discrimination and calibration ability (Fig. 3c, d, e, f). Finally, we explored the impact of PMV on postoperative short-term outcomes (Table 3), patients in the PMV group had a longer ICU length of stay, longer postoperative hospital length of stay and higher hospitalization costs. In addition, although he incidence of AKI and AHI was not significantly different between two groups, the PMV group is nominally higher. These clinical outcomes of PMV are all consistent with previous research [11, 12, 29, 30, 51].

The PMV nomogram can optimize clinical practice and also be used regularly by healthcare workers including nurses, clinicians, and social workers in clinical routine. It consists of five routinely collected clinical variables that are both easy to obtain, these variables include three preoperative variables and two postoperative variables, making it simple and convenient to use beside the bed. The application of PMV nomogram can further optimize clinical practice, clinicians can screen out the potential high-risk PMV children before surgery through three preoperative variables, make a detailed preoperative discussion, optimize anesthesia scheme and improve the modifiable intraoperative factors including shortening the CPB time and maintaining appropriate CVP as much as possible to further reduce the risk of PMV. Once patients access to ICU, ICU clinicians can make a fast scoring and risk prediction to quickly distinguish the high-risk PMV children and set up individualized medical managements for them, including changing ventilation strategy, preventive anti-infective therapy, positive organ function support and so on. Through the PMV nomogram, clinicians can make a rational risk stratification to help children early weaning from the ventilator, optimize the allocation of medical resources, shorten the ICU and hospital stay time, decline associated complications, lighten the ICU workload and further reduce unnecessary hospitalization costs.

### Limitations

The study still has several limitations. Firstly, due to the nature of the retrospective study, some detailed parameters about echocardiography, cardiac MRI and other laboratory tests are not available in many patients, these data were not routinely reported in Fuwai Hospital. Secondly, this was a single-center study, which could cause selection bias and our findings may not apply to other cardiac centers. In addition, until now there is no consensus on the definition of PMV for pediatric cardiac surgery, the threshold of 24 hour was based on The Society of Thoracic Surgeons. Thirdly, due to the low prevalence of EA, the sample size is relatively small, we conducted an internal validation by bootstrapping, it still exhibits a good discrimination. Despite this, to ensure the stability of the nomogram, it was better to be verified by an external validation. Lastly, we only compared the short-term outcomes and did not make a long-term follow-up for EA patients, the causal relationship between PMV and poor outcome still needs to be further explored in future research.

## Conclusion

The nomogram of PMV includes five routinely collected clinical variables that are both easy to obtain and can offers a simple-to-use tool to predict the risk of PMV for children after EA surgery. Clinicians can utilize it to predict the risk of PMV for children after EA corrective surgery. It can assist clinicians to make preoperative optimization and medical decision, decline associated poor outcomes, optimize medical resource allocation and reduce medical expenses.

## Supplementary Information

**Additional file 1.** STROBE Statement (checklist of items that should be included in reports of observational studies).

**Additional file 2.** Univariable logistic regression analyses of risk factors for prolonged mechanical ventilation.

## Data Availability

The datasets generated and/or analysed during the current study are not publicly available due data protection policy in our hospital but are available from the corresponding author on reasonable request.

## Abbreviations

ACC: Aortic cross-clamp
AHI: Acute hepatic injury
AKI: Acute kidney injury
AUC: Area under the curve
CI: Confidence interval
C/R: Cardiothoracic ratio
CPB: Cardiopulmonary bypass
CVP: Central venous pressure
EA: Ebstein anomaly
ICU: Intensive care unit
LVEDDz: Left ventricular end-diastolic diameter z-score
MAP: Mean arterial blood pressure
MV: Mechanical ventilation
PMV: Prolonged mechanical ventilation
ROC: Receiver operating characteristic
RV: Right ventricular
SpO2: peripheral oxygen saturation
TVP: Tricuspid valvuloplasty
TR: Tricuspid regurgitation
TVR: Tricuspid valve replacement
