# Supplementary material for: A simple-to-use nomogram for predicting prolonged mechanical ventilation for children after Ebstein anomaly corrective surgery: a retrospective cohort study
Source: BMC Anesthesiol. 2023 Jan 14;23:24. doi: 10.1186/s12871-022-01942-9 (PMC9839444; doi:10.1186/s12871-022-01942-9)
Supplement: Supplementary file 1 — Additional file 1. STROBE Statement (checklist of items that should be included in reports of observational studies). [file 12871_2022_1942_MOESM1_ESM.docx]

**Additional file 1.**

**STROBE Statement (checklist of items that should be included in reports of observational studies)**

|  | **Item**  **No.** | **Recommendation** |
| --- | --- | --- |
| **Title and abstract** | 1 | (a)Study design: a retrospective cohort study.  (b)Informative and balanced summary: Prolonged mechanical ventilation (PMV) after pediatric cardiac surgery imposes a great burden on patients in terms of morbidity, mortality as well as financial costs. This study aimed to establish a simple-to-use nomogram to predict the risk of PMV for EA children. This retrospective study included patients under 18 years who underwent corrective surgeries for EA. After multivariable regression, we obtained five risk factors of PMV and integrated them into a nomogram, the nomogram showed a good performance with an AUC of 0.805 and discriminative ability with corresponding Hosmer-Lemeshow p values > 0.05. It is a practical tool to early identify children at high-risk for PMV after EA corrective surgery. |
| **Introduction** |  |  |
| **Background/rationale** | 2 | For pediatric cardiac surgery, earlier studies have proved that patients with PMV are related to a higher mortality and morbidity compared with non-PMV. Early and accurate identification of children at high-risk for PMV could help clinicians to enact better individualized medical management, improve prognosis and allocate medical resources rationally. However, because of the rarity of the EA disease, there is no established scoring model used to identify high-risk PMV for EA children. |
| **Objectives** | 3 | The purpose of the study was to evaluate the incidence rate and independent risk factors of PMV for EA children, and construct a nomogram to early identify children at high risk for PMV after EA corrective surgery. |
| **Methods** |  |  |
| **Study design** | 4 | A retrospective cohort study |
| **Setting** | 5 | We retrospectively collected patients(age<18y) who underwent EA corrective surgery in Fuwai hospital from January 2009 to November 2021. Three independent medical students recorded the clinical data through electronic medical records and two independent researchers checked the data. |
| **Participants** | 6 | **Inclusion criteria:**1) under 18 years old; 2) undergone EA corrective operations from January 2009 to November 2021.  **Exclusion criteria**:1) missing data with postoperative MV time; 2) combined with confounding factors including preoperative tracheal intubation or tracheotomy, preoperative pulmonary infection, palliative surgery, complex cardiac malformations; 3) emergency surgery, surgical re-exploration and bedside thoracotomy; 4) severe postoperative complications.  Based on The Society of Thoracic Surgeons, patients with postoperative mechanical ventilation longer than 24 hours were included in the PMV group, and the others in the Non-PMV group. Finally, 44 patients were in the PMV group and 173 patients were in the Non-PMV group. |
| **Variables** | 7 | **Primary Outcome:** PMV (postoperative mechanical ventilation time more than 24 hours)  **Secondary Outcomes:** the duration of ICU stay, hospital stay and postoperative hospital stay, acute kidney injury, acute hepatic injury and hospitalization cost. |
| **Data sources**  **/ measurement** | 8 | **Data sources:** Electronic medical records.  **Measurement:**  PMV was defined as patients with postoperative mechanical ventilation longer than 24 hours. The preoperative SpO2 was collected when patient inhaled the air at admission to hospital.  The degree of TR was graded as none, mild, moderate, moderate-to-severe and severe on echocardiographic analysis.  The calculation of LVEDDz is available online: <http://parameterz.blogspot.com/2008/09/m-mode-z-scores.html>.  The MAP and CVP were the first measurement after 30 min of admission into pediatric ICU. The hospital stay time was defined as the duration from admission to discharge.  The postoperative hospital stay time was defined as the duration from the end of surgery to discharge.  AKI was defined as postoperative creatinine level more than 1.5-fold the baseline level.  AHI was defined as postoperative aspartate aminotransferase or alanine aminotransferase more than 2 times the upper limit and excluded AST increased within 24 hours after operation. |
| **Bias** | 9 | We collected the demographics, laboratory tests, and surgical associated information that could affected mechanical ventilation time, we put the clinically relevant variables and variables with P<0.1 in univariable regression into multivariable regression model by forward variable selection to control the potential confounding factors. |
| **Study size** | 10 | For the multivariable logistic model, we conducted a sample size calculation that could produce a small prediction error in the target population: <https://mvansmeden.shinyapps.io/BeyondEPV/>. n is the sample size, $\phi$is the anticipated outcome proportion (≤0.5), P is the number of candidate predictor parameters (≤30); MAPE refers to the average error of the allowable estimated outcome probability in the model. Considering that ebstein anomaly is a rare disease with a small sample size, we set MAPE as 0.1, P as 8 and $\phi$ as 0.2; finally, this study requires at least 160 participants (about 32 expected events) |
| **Quantitative variables** | 11 | Based on The Society of Thoracic Surgeons, patients with postoperative mechanical ventilation longer than 24 hours were included in the PMV group, and the others in the Non-PMV group. |
| **Statistical methods** | 12 | **(a)** The normally distributed continuous variables were described as the means ± standard deviation and compared by the Student’s t-tests. For non-normally distributed continuous variables, they were presented as the median with 25-75 percentiles and analyzed by Mann-Whitney U tests. Categorical variables were expressed as absolute (n) and relative (%) frequency, they were analyzed by Chi-squared test. We performed univariable and multivariable logistic regression analyses to obtain the independent risk factors for PMV. Collinearity relationships between risk factors were conducted before multivariate analysis by tolerance and VIF. Tolerance <0.1 or VIF>10 indicated that collinearity exist between variables. All variables with P<0.1 in univariable regression and the clinically relevant variables were put into a multivariable regression model by forward variable selection. We assessed the calibration and discrimination ability of the nomogram by the calibration curve, Hosmer-Lemeshow goodness-of-fit test and ROC curve. DeLong’s method was used to compare the areas under ROC curves (AUCs)[27]. To reduce confounding effect of age and surgical procedure, we conducted the subgroup analysis of age ≤ 6 years old and TVP groups*. P* value<0.05 was considered statistically significant. All statistical analysis was conducted in SPSS software version 25 (IBM, Armonk, NY, USA) and R software (version 3.2.0).  **(b)**there were no missing data. |
| **Results** |  |  |
| **Participants** | 13 | (a)There were 278 children who underwent EA corrective surgeries at Fuwai Hospital in the past 13 years.  (b)We excluded patients with a) missing postoperative MV time data (n=0); b)confounding factors impacting postoperative MV time (n=53); c) surgery associated factors(n=4) and d) postoperative complications (n=4).  (c)Finally, 217 patients were enrolled in our study, of which 44 patients were in the PMV group and 173 patients were in the Non-PMV group.  (d)A recruitment figure has been added (**Figure 1**). |
| **Descriptive data** | 14 | (a)The median age at surgery of the whole cohort was 6.1 (3-11.7) years, 123 (56.7%) patients were male, and 30(13.8%) patients had a C/R greater than 0.65. Most patients had associated cardiac anomalies of which the most common were atrial septal defect and patent foramen ovale, which were respectively present in 81(37.3%) and 68 (31.3%) of the patients. With regard to the echocardiographic parameters, the median ejection fraction was 66%(62.5%,70%), LVEDDz was -1.87(-2.79, -1.06) and aortic annular diameter was 15mm, TR more than moderate was present in 72.4% of patients and Carpentier type C or D was present in 20.3% of patients.. All surgeries were performed under CPB, with a median CPB time of 107 (89-136) minutes and aortic cross-clamp time of 74 (62-94) minutes.  (b)**Table 1** presents a summary of demographic data and perioperative variables of all patients.  (c)There were no missing data. |
| **Outcome data** | 15 | 44 patients were in the PMV group and 173 patients were in the Non-PMV group.  Compared with the Non-PMV group, patients in the PMV group had smaller weight and height, lower preoperative SpO2, larger C/R, longer CPB and ACC time, and higher CVP (**Table 1**). As for in-hospital outcome, Patients in the PMV group had higher incidence of reintubation, longer ICU stay time, longer postoperative hospital stay time and higher hospitalization costs (**Table 3**). |
| **Main results** | 16 | According to the univariable and multivariable regression results, we found that preoperative SpO2, C/R>0.65, Carpentier type C or D, CPB time and CVP were independent risk factors for PMV. The odd ratios and 95% confidence intervals (CI) of risk factors for PMV are shown in **Table 2**. Then we incorporated the independent risk factors into a nomogram of PMV in **Figure 2**. The nomogram showed a good calibration and discrimination ability with AUC of 0.805 and the corresponding Hosmer-Lemeshow p values > 0.05 (**Figure 3a and Figure 3b)**. |
| **Other analyses** | 17 | We applied the nomogram into subgroups, the AUCs of nomogram in the subgroups with age ≤ 6 years old and TVP subgroup were 0.809 and 0.807 (**Figure 3c,3e**). The corresponding calibration curves of subgroups are respectively shown in **Figure 3d** and **Figure 3f**. It still showed acceptable discrimination and calibration ability. |
| **Discussion** |  |  |
| **Key results** | 18 | The nomogram of PMV includes five routinely collected clinical variables that are both easy to obtain and can offers a simple-to-use tool to predict the risk of PMV for children after EA surgery. Clinicians can utilize it to predict the risk of PMV for children after EA corrective surgery. It can assist clinicians to make preoperative optimization and medical decision, decline associated poor outcomes, optimize medical resource allocation and reduce medical expenses. |
| **Limitations** | 19 | Firstly, due to the nature of the retrospective study, some detailed parameters about echocardiography, cardiac MRI and other laboratory tests are not available in many patients, these data were not routinely reported in Fuwai Hospital. Secondly, this was a single-center study, which could cause selection bias and our findings may not apply to other cardiac centers. In addition, until now there is no consensus on the definition of PMV for pediatric cardiac surgery, the threshold of 24 hour was based on The Society of Thoracic Surgeons. Thirdly, due to the low prevalence of EA, the sample size is relatively small. Lastly, we only compared the short-term outcomes and did not make a long-term follow-up for EA patients, the causal relationship between PMV and poor outcome still needs to be further explored in future research. |
| **Interpretation** | 20 | PMV is a common complication of cardiac surgery; in our cohort, approximately 20% of children were ventilated more than 24 hours after EA corrective surgery. Until now no risk scoring model of PMV for EA children has been explored because of the rarity and various modes of presentation of EA. We are the first to develop a nomogram to predict the risk of PMV for children with EA using routinely collected clinical variables. This nomogram is composed with five routine data including SPO_2_, C/R, Carpentier type, CPB, and CVP. It is simple for bedside use, clinicians could quickly distinguish potential children at high-risk for PMV and impose personal medical intervention. By this nomogram, clinicians can make a rational risk stratification to help children early weaning from the ventilator, shorten the ICU and hospital stay time, decline associated complications, lighten the ICU workload and further reduce unnecessary hospitalization costs. |
| **Generalisability** | 21 | The findings came from a single-center study, due to the low prevalence of EA, the sample size is relatively small, we conducted an internal validation by bootstrapping, it still exhibits a good discrimination. However, to ensure the stability of the nomogram, it was better to be verified by an external validation. |
| **Other information** |  |  |
| **Funding** | 22 | There were no funding supporting in this study. |
