# Supplementary material for: A simple-to-use nomogram for predicting prolonged mechanical ventilation for children after Ebstein anomaly corrective surgery: a retrospective cohort study
Source: BMC Anesthesiol. 2023 Jan 14;23:24. doi: 10.1186/s12871-022-01942-9 (PMC9839444; doi:10.1186/s12871-022-01942-9)
Supplement: Supplementary file 2 — Additional file 2. Univariable logistic regression analyses of risk factors for prolonged mechanical ventilation. [file 12871_2022_1942_MOESM2_ESM.docx]

**Additional file 2**

**Univariable logistic regression analyses of risk factors for prolonged mechanical ventilation.**

| Variable | OR(95% CI) | *P* value |
| --- | --- | --- |
| Demographics |  |  |
| Age (years) | 0.964(0.902,1.03) | 0.276 |
| Gestational  age (weeks) | 0.895(0.701,1.142) | 0.373 |
| male | 1.274(0.647,2.507) | 0.483 |
| Weight (kg) | 0.988(0.97,1.006) | 0.178 |
| Height (cm) | 0.99(0.98,1.001) | 0.071 |
| BMI (kg/m^2^) | 0.938(0.845,1.04) | 0.222 |
| Preoperative data |  |  |
| SpO2(%) | 0.88(0.816,0.948) | 0.001 |
| NYHA class  (III/IV) | 1.856(0.712,4.837) | 0.205 |
| C/R>0.65 | 2.702(0.392,2.707) | 0.019 |
| WPW syndrome | 1.03(0.392,2.707) | 0.953 |
| Associated cardiac defects |  |  |
| ASD | 1.209(0.615,2.377) | 0.583 |
| PDA | 4.071(0.557,29.749) | 0.166 |
| PFO | 0.783(0.375,1.635) | 0.516 |
| Ultrasound parameter |  |  |
| LVEF (%) | 1.006(0.955,1.06) | 0.817 |
| TR>moderate | 1.934(0.841,4.445) | 0.12 |
| Carpentier type C+D | 4.499(2.164,9.353) | <0.001 |
| LVEDDz | 0.96(0.781,1.181) | 0.701 |
| RVAD (mm) | 1.015(0.988,1.043) | 0.277 |
| SLD (mm) | 0.991(0.964,1.018) | 0.505 |
| PLD (mm) | 0.994(0.973,1.014) | 0.544 |
| AOAD (mm) | 0.913(0.825,1.01) | 0.076 |
| Preoperative biomarkers |  |  |
| WCC (10^9^/L) | 0.995(0.943,1.049) | 0.851 |
| RBC(10^12^/L) | 1.018(0.561,1.847) | 0.952 |
| PLT (10^9^/L) | 0.998(0.993,1.002) | 0.347 |
| Hb(g/L) | 1.005(0.989,1.022) | 0.558 |
| Hct (%) | 1.018(0.96,1.081) | 0.545 |
| CK-MB (IU/L) | 0.984(0.962,1.007) | 0.175 |
| hs-CRP(mg/L) | 1.004(0.847,1.191) | 0.962 |
| Intraoperative data |  |  |
| TVR | 1.6(0.3,8.536) | 0.582 |
| CPB time (min) | 1.015(1.006,1.023) | <0.001 |
| ACC time (min) | 1.012(1.002,1.023) | 0.019 |
| T_min_ (℃) | 0.927(0.72,1.193) | 0.557 |
| Dexamethasone | 0.471(0.24,0.924) | 0.029 |
| Ulinastatin | 1.049(0.527,2.089) | 0.891 |
| Infusion volume (ml) | 1(0.998,1.001) | 0.609 |
| Blood loss (ml) | 1(0.998,1.001) | 0.617 |
| CVP (mmHg) | 1.202(1.07,1.352) | 0.002 |
| MAP (mmHg) | 0.995(0.969,1.022) | 0.722 |

Quantitative variables are expressed as means ± SD or median (interquartile range); Categorical variables are expressed as frequency (percentage). BMI, body mass index; SpO2, peripheral oxygen saturation; NYHA class, New York Heart Association Classification; C/R, cardiothoracic ratio; WPW, Wolff-Parkinson-White; ASD, atrial septal defect; PDA, patent ductus arteriosus; PFO, patent foramen ovale; LVEF, left ventricular ejection fraction; TR, tricuspid regurgitation; LVEDDz, Left ventricular end-diastolic diameter z-score; RVAD, right ventricular anteroposterior diameter; PLD, Posterior leaflets displacement ; SLD, Septal leaflets displacement ; AOAD, Aortic annular diameter ; WCC, white blood cell; RBC, red blood cell; PLT, platelet; Hb, Hemoglobin; Hct, hematocrit; CK-MB, isoenzyme of creatine kinase-MB; hs-CRP, C-reactive protein; TVR, tricuspid valve replacement; CPB, cardiopulmonary bypass; ACC, aortic cross-clamp; T_min_, the minimum temperature; CVP, central venous pressure; MAP, mean artery blood pressure.
